# Mitochondrial DNA D-Loop Diversity of the Helmeted Guinea Fowls in Kenya and Its Implications on HSP70 Gene Functional Polymorphism

**DOI:** 10.1155/2018/7314038

**Published:** 2018-11-13

**Authors:** Philip Murunga, Grace Moraa Kennedy, Titus Imboma, Phillista Malaki, Daniel Kariuki, Emmanuel Ndiema, Vincent Obanda, Bernard Agwanda, Jacqueline Kasiiti Lichoti, Sheila Cecily Ommeh

**Affiliations:** ^1^Institute for Biotechnology Research (IBR), Jomo Kenyatta University of Agriculture and Technology (JKUAT), P.O. Box 62000, City Square 00200, Nairobi, Kenya; ^2^Department of Zoology, National Museums of Kenya, Kenya; ^3^Department of Biochemistry, Jomo Kenyatta University of Agriculture and Technology, Kenya; ^4^Department of Earth Sciences, National Museums of Kenya, Kenya; ^5^Department of Veterinary Services, Kenya Wildlife Service, Kenya; ^6^Directorate of Veterinary Services, State Department of Livestock, Ministry of Agriculture, Livestock, Fisheries and Irrigation, Kenya

## Abstract

We analyzed variations in 90 mitochondrial DNA (mtDNA) D-loop and heat shock protein 70 (HSP70) gene sequences from four populations of domesticated helmeted Guinea fowls (70 individuals) and 1 population of wild helmeted Guinea fowls (20 individuals) in Kenya in order to get information about their origin, genetic diversity, and traits associated with heat stress. 90 sequences were assigned to 25 distinct mtDNA and 4 HSP70 haplotypes. Most mtDNA haplotypes of the domesticated helmeted Guinea fowls were grouped into two main haplogroups, HgA and HgB. The wild population grouped into distinct mtDNA haplogroups. Two mtDNA haplotypes dominated across all populations of domesticated helmeted Guinea fowls: Hap2 and Hap4, while the dominant HSP70 haplotype found in all populations was CGC. Higher haplotype diversities were generally observed. The HSP70 haplotype diversities were low across all populations. The nucleotide diversity values for both mtDNA and HSP70 were generally low. Most mtDNA genetic variations occurred among populations for the three hierarchical categories considered while most variations in the HSP70 gene occurred among individuals within population. The lack of population structure among the domestic populations could suggest intensive genetic intermixing. The differentiation of the wild population may be due to a clearly distinct demographic history that shaped its genetic profile. Analysis of the Kenyan Guinea fowl population structure and history based on mtDNA D-loop variations and HSP70 gene functional polymorphisms complimented by archaeological and linguistic insight supports the hypothesis that most domesticated helmeted Guinea fowls in Kenya are related to the West African domesticated helmeted Guinea fowls. We recommend more molecular studies on this emerging poultry species with potential for poverty alleviation and food security against a backdrop of climate change in Africa.

## 1. Introduction

The helmeted Guinea fowl (*Numida meleagris*) is a terrestrial game bird that is widespread and abundant in Africa. It is found in a wide range of sub-Saharan, open country vegetation type [1, 2]. Guinea fowls are a ready source of animal protein (meat and eggs) and income as well as a source of manure for soil enrichment [3–5]. Their lean meat with its characteristic flavor is relished by the local population in Kenya [6].

The mitochondrial DNA (mtDNA) is a circular molecule that is 16,726 base pairs in size in Guinea fowls [7] and has a maternal mode of inheritance [8]. It is relatively easy, rapid, and inexpensive to sequence, and research work on rapidly evolving loci provides sufficient variation to draw inferences on the structure of populations [9–12]. The control region, also referred to as D-loop, often mutates faster than the rest of the mtDNA [12, 13] and appears to be highly variable in birds [12, 14]. Analysis of polymorphism in the D-loop region has proved to be informative in previous studies on genetic variation, structure, and phylogeography in birds [12, 15–19]. The study of the genealogical origin of Guinea fowls was first undertaken by Kimball [20] who examined the phylogenetic position of three species of peafowl in the family* Phasianidae *in relation to the helmeted Guinea fowl in the family* Numididae,* using mtDNA D-loop and cytochrome b sequences. In their examination, Kimball [20] showed that the three peafowl species formed a monophyletic clade and that peafowl were genetically separated from Guinea fowls in the phylogenetic tree. Additionally, work on mitochondrial DNA variation of domesticated helmeted Guinea fowls in Nigeria revealed a lack of genetic differentiation within most Nigerian domesticated helmeted Guinea fowl which was attributed to intensive genetic admixture [21].

Heat stress in birds is one of the main concerns in poultry farming since it causes high mortality and low productivity especially during the hottest seasons [22, 23]. In response to thermal stress in the tissues of living animals, cells synthesize heat shock proteins (HSPs) of low molecular weight that have specific functions in cell growth and in reversing or preventing damage caused by stress [23]. Among the HSPs, HSP70 shows the highest levels under stressful conditions [23]. The HSP70 is therefore a useful molecular marker for studying environmental stress inpoultry. Studies on heat shock protein 70 genes in chicken revealed that only the expression of HSP70 (*Nm*HSPA2 in Guinea fowl) is promoted by heat shock [24, 25]. Other findings on HSP70 in Japanese quail from Brazil revealed alterations in the DNA sequences with the appearance of a possible polymorphism [23]. Gaviol [23] suggested that there was need to study this polymorphism to determine if it had any association with heat resistance.

This study aimed to analyze variations in the mtDNA D-loop and HSP70 gene of the helmeted Guinea fowl in Kenya for the first time, in order to get some initial information about their genetic diversity, possible genetic structure, and functional polymorphisms in HSP70 that could be associated with heat stress.

## 2. Materials and Methods

### 2.1. Sample Collection

**Figure 1:**
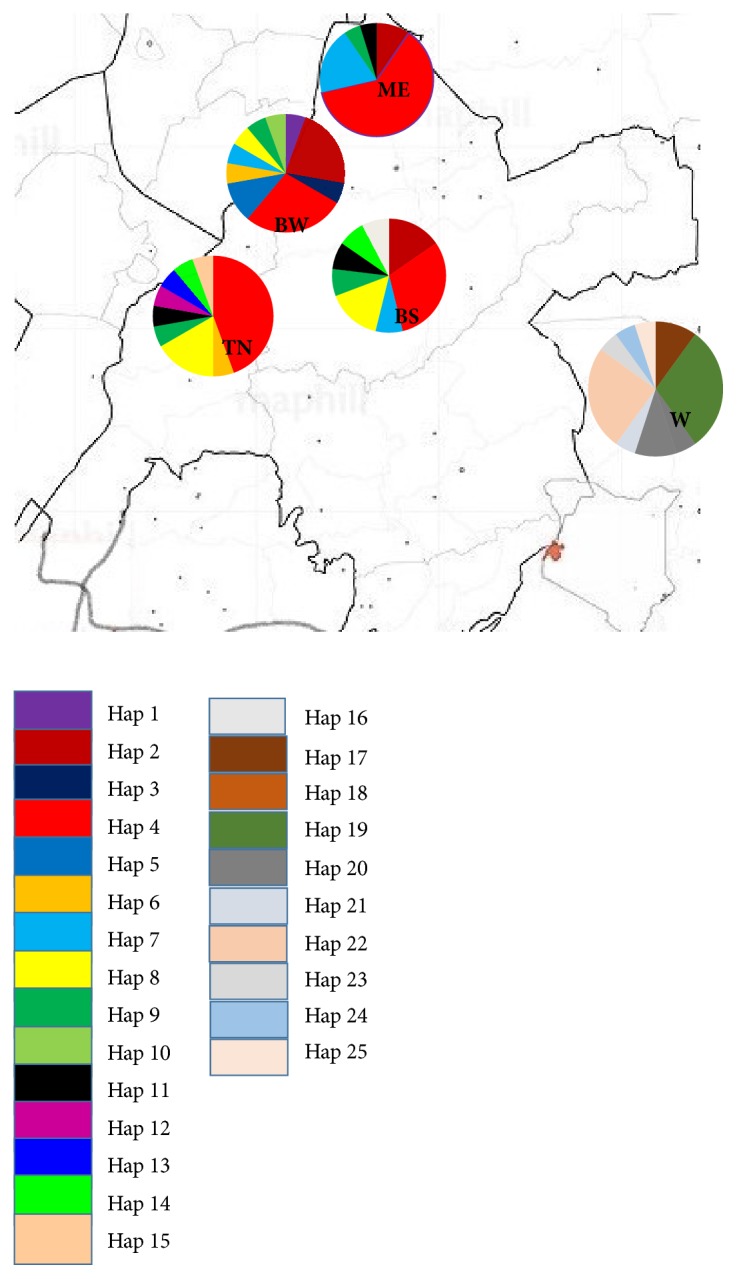
Pie diagrams showing sampled areas and the distribution of mtDNA haplotypes in helmeted Guinea fowl populations in Kenya. Different colors indicate specific haplotypes. Initials indicate the populations sampled; BW represents Bungoma West, TN Teso North, ME Mt. Elgon, and W the wild Guinea fowls which were sampled in Laikipia (source: maphill.com).

This study received ethical clearance from the Kenya Wildlife Service under permit number KWS/BRM/5001 to sample wild Guinea fowls and a “no objection for the research” from the Directorate of Veterinary Services, Ministry of Agriculture, Livestock and Fisheries in Kenya under permit number RES/POL/VOL.XXVII/162 to sample domestic Guinea fowls. Blood samples were collected from 90 unrelated adult individuals from five populations in Kenya (Figure 1). Four of these populations are located in Western Kenya: Teso North (n=18), Bungoma South (n=13), Bungoma West (n=18), and Mt. Elgon (n=21). The fifth population (n=20) consisting of wild birds was sampled from two sanctuaries in Laikipia: Mpala Research Centre and Mt. Kenya Game Range. Genomic DNA was extracted from air-dried blood preserved on FTA classic cards (Whatman Biosciences) using the manufacturers' protocol. To ascertain the genetic affinities of the study population to helmeted Guinea fowls from other African countries, 241 sequences were downloaded from the Genbank and were included in the analysis.

### 2.2. PCR Amplification and Sequencing

The first 700 bp of the mtDNA D-loop region was amplified via PCR using the forward primer AVIF2 and reverse primer CR1b [29]. PCR amplifications were carried out in 25 *μ*l reaction volumes containing 20 ng genomic DNA, 1 X PCR buffer (10 mM Tris–HCl pH 8.3, 50 mM KCl, 0.1% Triton X-100), 2.5 mM of each dNTP, 10 pM of each primer, and 1 unit of Taq DNA polymerase (Promega, Madison WI, USA). Thermocycling conditions were as follows: 94°C (3 min), 35 cycles of 94°C (1 minute), 58°C (1 min), 72°C (2 min), and a final extension step at 72°C (10 min). The* Gallus gallus *HSP70 ortholog in* Numida meleagris*, that is* Nm*HSPA2, was amplified via PCR using the forward primer HSP70-F 5′-ATCATCGCCAATGACCAGGG-3′ (20) and reverse primer HSP70-R 5′-CATTCTTCTCTCCAGCCCGG-3′ (20). PCR amplifications were performed in a 10 *μ*l reaction volume containing 3.8 *μ*l of double distilled water, 1 *μ*l of template genomic DNA, 5 *μ*l of Thermo Scientific™ DreamTaq™ Green Master Mix (2X), and 0.2 *μ*l of 20pM/ *μ*l primer (forward and reverse). The PCR was run under the following conditions: One cycle of initial denaturation at 94°C (3 min), 30 cycles of 94°C for 30 seconds, 55°C for 30 seconds annealing, 72°C for 30 seconds for primer extension, and a final extension step at 72°C (7 min).

PCR products were purified using the Wizard® SV gel and PCR Clean-Up Kit (Promega, Madison WI, USA). Purified products were sequenced directly using the Big Dye® Terminator v3.1 (Applied Biosystems, USA) on an ABI prism 3730 Avant DNA analyzer. The relevant PCR primers were used for the sequencing reactions.

### 2.3. Data Analysis

The sequences generated were edited manually using Chromas Lite version 2.1.1 [29]. The consensus sequences were then aligned using ClustalX version 2.1 [30] against reference sequences from Genbank. Subsequent analyses were restricted to the first 351-353 bp of mtDNA D-loop incorporating the first hyper variable segment (HVS1) and a 508 bp promoter region of HSP70.

Construction of the haplotypes was done both manually and by use of DnaSP v5.10 [31]. Genetic diversity indices for each population were calculated using DnaSP v5.10 on both mtDNA and HSP70 and ARLEQUIN v3.5.1.2 [32]. For demographic analysis, we computed the distribution of the observed pairwise nucleotide differences (mismatch distribution) and the expected values for no recombination in growing-declining populations [33] using ARLEQUIN v3.5.1.2. ARLEQUIN tests the goodness-of-fit to this model by SSD test statistic (the sum of squared differences between the observed and the expected mismatch distributions). DnaSP v5 calculates Tajima's D [34], Fu's F_s_ [35], and R_2_ [36] statistics and estimates their significance using a coalescent simulation algorithm. We used 1,000 replications in these coalescent simulations.

The number of haplogroups was determined by constructing a median joining (MJ) network [26] using NETWORK v5.0.0.0. For HSP70 gene, a phylogenetic tree involving the haplotypes observed was constructed using the Maximum Likelihood algorithm as implemented in MEGA v6.06 following 1,000 bootstrap replications [37]. To portray the affinity of Kenyan mtDNA haplotypes to those observed in other parts of Africa, an MJ network incorporating haplotypes downloaded from Genbank (Table S1) was constructed. The nomenclature of the haplogroups observed in this study were compared to the study of Adeola et al. [21].

To infer the mtDNA maternal genetic structure of helmeted Guinea fowls across Kenya, analysis of molecular variance (AMOVA) was performed using ARLEQUIN. To assess the nonrandom association between genetic differentiation (F_ST_) and geographic distances between populations, a Mantel test was used to plot the regression graph of the genetic and geographic distances using GenAlEx v6.501 software [27] which is a Microsoft Excel add-in.

## 3. Results and Discussion

### 3.1. Mitochondrial DNA D-Loop Variation and Haplotype Distribution Pattern

We obtained partial 351-353 bp mitochondrial DNA D-loop sequences from samples of 90* Numida meleagris* individuals captured in Kenya. The sequences were compared with those obtained from Genbank having accession numbers KP218263-KP218503 [21] and AP005595 [7]. The vulturine Guinea fowl accession number NC_014180 [38] was included as an outgroup.

We identified 25 unique haplotypes (Hap1-Hap25) defined by 41 polymorphic sites. For this study, the individual haplotypes were abbreviated Hap followed by a Hindu-Arabic numeral. These have been deposited into Genbank under accession numbers MH703540- MH703564. We observed an insertion in all individuals in the wild population and a second insertion in two wild individuals. The frequencies of the observed haplotypes in the various populations are represented in a pie diagram (Figure 1) and also shown in Table S2 in the Supplementary Materials provided separately. In agreement with a previous study on Nigerian helmeted Guinea fowls [21], we observed two major haplotypes, Hap4 and Hap2. The 16 haplotypes observed in the four populations with domesticated individuals compare favorably with the 19 haplotypes identified in Nigerian domesticated helmeted Guinea fowls [21]. Most of the Nigerian, Kenyan, and Chinese domesticated helmeted Guinea fowls also belong to haplotypes Hap2 and Hap4, which strongly suggests possibility of a common origin of both the Kenyan domesticated helmeted Guinea fowls and West African domesticated helmeted Guinea fowls derived from the West African* Numida meleagris galeata *[39]. We also observed that the 9 haplotypes identified in the wild helmeted Guinea fowls were not shared by the domesticated helmeted Guinea fowls. Their unique haplotype could be a consequence of unique demographic histories that have shaped their haplotype profiles [21]. Using microsatellite markers to compare genetic variation between red jungle fowl and commercial chicken lines [40] and genetic variation between wild and domesticated helmeted Guinea fowl [41], it was shown that the wild populations genetically differed from the domesticated populations.

The extent of haplotype sharing indicates the absence of a population structure in Kenya's domesticated helmeted Guinea fowls. Muchadeyi [42] and Mtileni [43] proposed that large effective population sizes as well as continuous gene flow may be the forces responsible for the lack of population differentiation among the local chicken genotypes in their studies. Similarly, Weimann [41] attributed the lack of a clear population structure in domesticated helmeted Guinea fowl populations to large population sizes and continuous gene flow. It is interesting to note that a similar pattern of lack of phylogeographic structure in poultry chicken, such as domesticated helmeted Guinea fowl in Ghana [44] and Nigeria [21], from East Africa [29] and Nigeria [45] has been observed. This could likely be due to intensive genetic intermixing between populations due to human migration and trading [21, 46]. Hence the lack of genetic differentiation in Kenyan domesticated helmeted Guinea fowl may likewise be due to intensive genetic admixture. Adeola [21] however noted that short DNA sequences with inadequate sample size may result in insufficient genetic information to clearly infer the population structure.

We calculated several diversity indices for the five populations as shown in Table 1. The lowest haplotype diversities (h) are observed in the Mt. Elgon and Teso North populations. The other populations show higher haplotype diversity values. The nucleotide diversity (*π*) values are generally low, with the wild population showing highest nucleotide diversity. Avise [47] suggested that high levels of haplotype diversity could be due to large population sizes. These results are similar to those observed in Nigerian helmeted Guinea fowls [21] where the lowest haplotype diversity was 0.529±0.095 and the highest haplotype diversity was 0.821±0.082. The low nucleotide diversity values indicate that the observed haplotypes were closely related [12]. The low haplotype diversities observed in the Mt. Elgon and Teso North could be attributed to recent domestication (<5,000 years ago) from a small founder population [45]. Insufficient time may have passed since domestication to allow for the accumulation of mutations. Additionally, the rearing system in most of the households keeping these poultry species encourages inbreeding since they usually start with two related birds, a male and female that are mostly siblings.

To reveal the historical population dynamics of the studied helmeted Guinea fowl populations across Kenya, we calculated observed and expected distributions of mismatches under the model of growing-declining populations [33] as shown in Figure S1 in the Supplementary Materials. The mismatch distribution pattern is multimodal.  Table S3 in the Supplementary Materials shows a summary of statistics about the demographic history of helmeted Guinea fowl populations in Kenya (simulated sum of squares differences or SSD, Tajima's D and Fu's F_s_). Our results show that all the sampled populations except Bungoma West have insignificant SSD values (P>0.05). Tajima's D (with positive values) and Fu's F_s_ (with mostly negative values) were not significant (P>0.05). This could be due to the small number of samples studied since Tajima's D has low power in detecting population expansion when the sample size is small [11]. Demographic and spatial expansion of the mtDNA haplotypes in the various populations are also shown (Table S4 in the Supplementary Materials). Harpending's demographic expansion raggedness index “r” [48] of the mtDNA haplotypes is significant for the Bungoma West (P=0.021) and Mt. Elgon (P=0.011) populations supporting a model of demographic expansion for these populations. However, the spatial expansion raggedness index of the mtDNA haplotypes is not significant (P>0.05) for all the five populations of helmeted Guinea fowls in Kenya. Like in previous studies that supported a model of demographic expansion over all East African chicken populations [29], these results support a model of demographic expansion of the Bungoma West and Mt. Elgon Guinea fowls. We also noted that Guinea fowls in Mt Elgon had the lowest number of haplotypes and haplotype diversity in comparison to the other Guinea fowls. Previous studies had shown low genetic diversity in domesticated Guinea fowl outside their area of origin, and this was attributed to a small founder population [21, 49] and many years of inbreeding. However, the raggedness index, Tajima's D, and Fu's F_s_ statistics do not support demographic and spatial expansion for mtDNA haplotypes across the other populations as previously suggested by Mwacharo [25].

**Figure 2:**
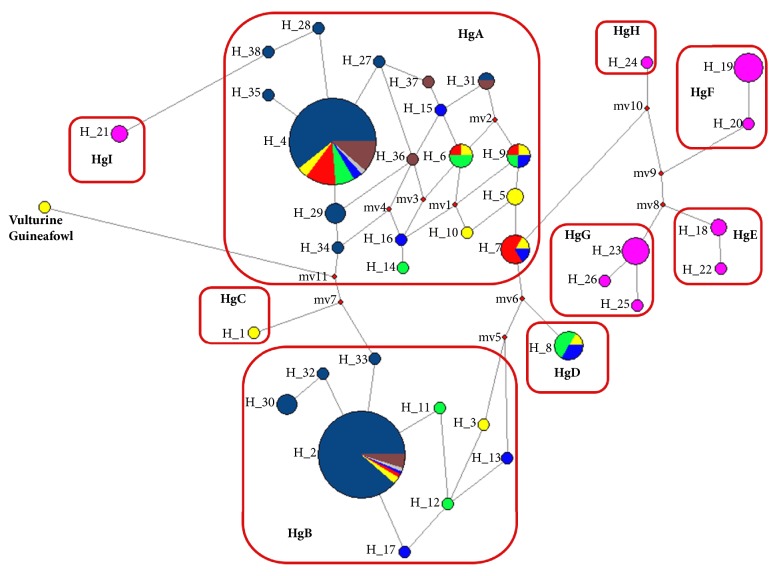
mtDNA of domesticated helmeted Guinea fowls in Nigeria, Kenya, and China [21] constructed using NETWORK v5.0.0.0 [26]. Pie diagrams show haplotypes, and colors indicate the populations sampled: yellow, Bungoma West; green, Teso North; red, Mt. Elgon; blue, Bungoma South; pink, wild; deep blue, Nigerian reference sequences; grey, Kenyan reference sequences; brown, Chinese reference sequences. Sizes of circles are proportional to frequencies and *m* is the number of mutation steps.* mv* is the median vector used to connect indirectly related haplotypes. The vulturine Guinea fowl was included as an outgroup.

Figure 2 shows a median joining network of the 90 helmeted Guinea fowl samples constructed using NETWORK v5.0.0.0 [26]. The median joining network shows that most of the domesticated helmeted Guinea fowls grouped into two major haplogroups named HgA and HgB in a previous study [21] clustered around Hap2 and Hap4. Most of the published sequences of Nigerian, Kenyan, and Chinese domesticated helmeted Guinea fowls [21] also group into haplogroups HgA and HgB, indicating a most probable common origin of both West African and Kenyan domesticated helmeted Guinea fowls. The 20 wild helmeted Guinea fowls are grouped into five distinct haplogroups named HgE, HgF, HgG, HgH, and HgI in this study. A very clearly distinct haplogroup HgI comprising two wild individuals was identified. The median joining network seems to suggest that haplogroup HgI has a closer genetic relationship with domesticated helmeted Guinea fowls than with other wild helmeted Guinea fowls. We note that the Guinea fowls in this haplogroup were sampled from farmers with close proximity to the forest; hence there was evidently an interaction among domesticated and wild Guinea fowls leading to gene flow between wild and domesticated helmeted Guinea fowls. The median vectors may represent either unsampled haplotypes, haplotypes never introduced into Kenya, or haplotypes introduced into Kenya but becoming extinct shortly upon arrival or later [25]. The star-like pattern exhibited in haplogroups HgA and HgB is an evidence of rapid population expansion [21]. The extent of haplotype sharing in the network between domesticated populations indicates the absence of population structure in Kenyan domesticated Guinea fowls as earlier discussed.

To infer the maternal genetic structure of helmeted Guinea fowls across Kenya, we performed analysis of molecular variance (AMOVA) as shown in Tables S5, S6, and S7 in the Supplementary Materials. Considering the five sampled regions as a hierarchical cluster, 51.54% of the genetic variation was observed among populations. This value, however, increases to 70.74% when wild helmeted Guinea fowls as a group are compared with the domesticated helmeted Guinea fowls and decreases to 57.02% when three groups, that is, Teso South and Mt. Elgon, Bungoma West and Bungoma South, and the wild Guinea fowls, are considered. Results from the three hierarchical categories therefore show that among-region distribution of variation is higher than within-region variation in the mitochondrial DNA D-loop region of helmeted Guinea fowls in Kenya.

**Figure 3:**
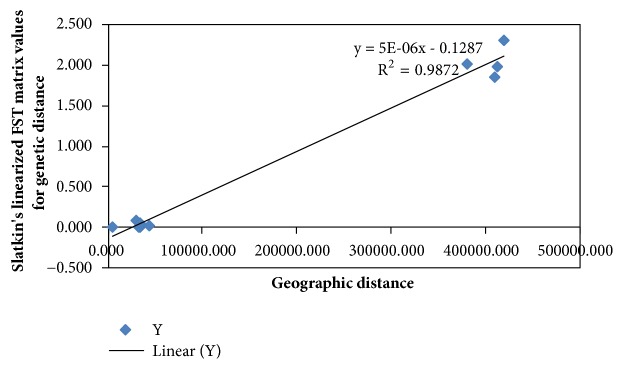
A regression graph showing the relationship between geographic and genetic distance matrices of helmeted Guinea fowls in Kenya, constructed by plotting the regression graph of the genetic and geographic distances using GenAlEx v6.501 software [27] which is a Microsoft Excel add-in.

The nonrandom association between genetic differentiation (F_ST_) and geographic distances between sampled regions was assessed using a Mantel test (Figure 3).

A strong and significant positive correlation (r = 0.9936, P>0.05) is observed between genetic variations and the geographic location in helmeted Guinea fowls in Kenya just like previously described by Mwacharo [25]. This contrasts with the findings of Ommeh [50] that showed a slight negative correlation between allele frequencies and the geographic location in indigenous village chicken populations. The Mantel test revealed lack of a population structure within Kenya's domesticated helmeted Guinea fowl mtDNA haplotypes.

### 3.2. HSP70 Gene Functional Polymorphisms

We obtained partial sequences of the helmeted Guinea fowl HSP70 gene of 508 bp in length from samples of 90 individuals captured in Kenya. Three of these sequences were of poor quality and were not considered for further analysis. The sequences are compared with HSP70 sequences of other related avian species downloaded from Genbank. We observed four haplotypes, TGC, TAC, TGT, and CGC, with three polymorphic sites (all transitions) shown in Table S8 in the Supplementary Materials. The haplotype sequences have been deposited into Genbank under accession numbers MH703565- MH703568.

The relative frequencies of the observed haplotypes in the various populations are shown in Table S9 in the Supplementary Materials. Haplotype TGC is shared in all the five populations. Unique mutations in the heat shock protein 70 gene in the wild helmeted Guinea fowl population (haplotypes TGT and CGC) are observed, which are not evident in the domesticated helmeted Guinea fowls. Again, an A/G transition (haplotype TAC) is observed in two domesticated individuals in the Teso North population that are not observed in all the other populations. A theoretical relationship between* Gallus gallus* HSP70 genotype and heat shock resistance (heat tolerance) has been proposed [51]. Individual variations in heat shock responses may be related to DNA polymorphisms in the HSP70 gene in birds [24, 52].

We calculated several diversity indices for the five populations as shown in Table 2. All the populations had low values of haplotype diversity (h) and nucleotide diversity (*π*). The wild population has the highest diversity indices.

Haplotype and nucleotide diversity values are generally low. These lower values reinforce our hypothesis that the observed haplotypes were closely related [12] and could be attributed to recent domestication [45] from a small founder population.

**Figure 4:**
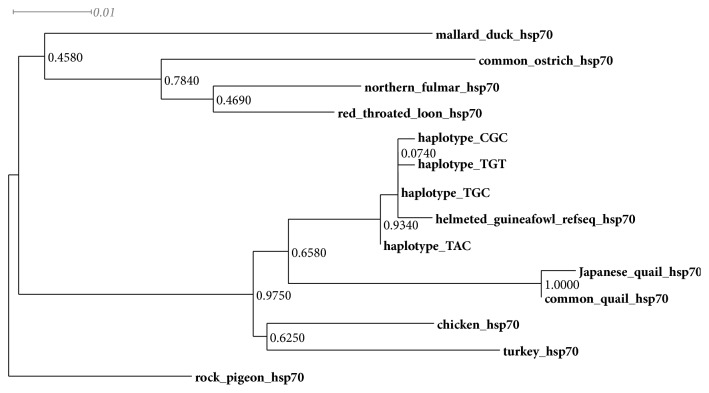
Phylogeny of the helmeted Guinea fowls constructed using Maximum Likelihood as implemented in MEGA v6.06 [7] with 1000 bootstrap replications. The model used was K2+G; gamma shape parameter is 0.1264. The rock pigeon was included as an outgroup.

The phylogenetic relationship of the various helmeted Guinea fowl haplotypes in Kenya is compared with HSP70 sequences of other avian species and shown in Figure 4. Phylogenetic analysis of the four HSP70 haplotypes with other avian HSP70 sequences downloaded from Genbank shows that all the haplotypes clustered together. We also observe that haplotype TAC seems to be more genetically distant from the other haplotypes. The helmeted Guinea fowl HSP70 phylogenetic tree reveals a strong relationship with HSP70 sequences of other* Galliformes*.

**Figure 5:**
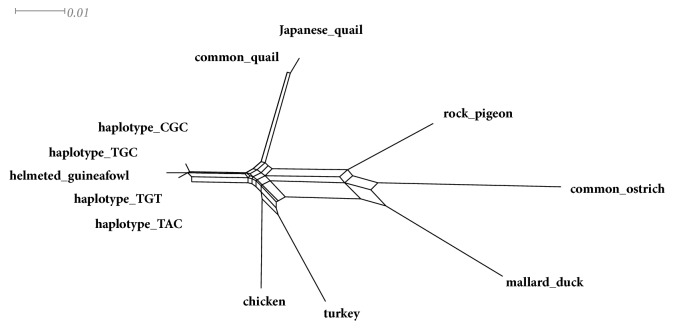
Phylogenetic network of the four helmeted Guinea fowl HSP70 haplotypes with other avian HSP70 sequences. The network was generated using SplitsTree version 4.13.1 [28].

The phylogenetic network diagrams produced using SplitsTree were used to validate the haplotypes. The splits decomposition network of the HSP70 haplotypes in Guinea fowls and related avian species is shown in Figure 5.

The splits decomposition network also reveals that all the haplotypes clustered together with haplotype TAC observed to be genetically distant in relation to the other haplotypes. The other avian species were also relatively distant from the four Guinea fowl HSP70 haplotypes.

To infer the population genetic structure of HSP70 haplotypes of helmeted Guinea fowls across Kenya, analysis of molecular variance (AMOVA) was performed (Tables S10 and S11 in the Supplementary Materials). When wild helmeted Guinea fowls as a group are compared against the domesticated helmeted Guinea fowls, 49.64% of the genetic variation was observed among individuals within population. This value increases to 56.98% when three groups are considered: Teso South and Mt. Elgon, Bungoma West and Bungoma South, and the wild population. Results from the two hierarchical categories show that most variations occurred among individuals within population in the HSP70 gene of helmeted Guinea fowls in Kenya.

**Figure 6:**
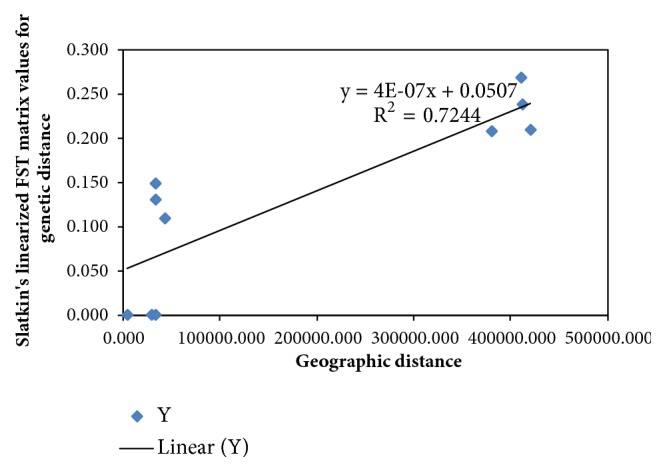
**A** regression graph showing the relationship between geographic and genetic distance matrices of helmeted Guinea fowl populations in Kenya, constructed by plotting the regression graph of the genetic and geographic distances using GenAlEx v6.501 software [27] which is a Microsoft Excel add-in.

A Mantel test was used to assess the nonrandom association between genetic differentiation (F_ST_) and geographic distances between populations by plotting the regression graph of the genetic and geographic distances (Figure 6).

From the results, a significant (P>0.05) and strong positive correlation is observed between genetic variation and the geographic location in helmeted Guinea fowl populations in Kenya as previously described by Mwacharo [25]. Again, the Mantel test reveals lack of a population structure within Kenya's domesticated helmeted Guinea fowl HSP70 haplotypes just like the mtDNA data revealed.

### 3.3. Archaeological and Linguistic Insight into the Origin of Helmeted Guinea Fowls

Previous analysis of Guinea fowl DNA indicates a possible* Numididae* divergence from the* Phasianidae* lineage some 38 million years ago [53]. Martinez [53] went on to suggest that Guinea fowls could have originated from the Savanna areas of Asia, having probably evolved from a francolin-like phasianid that colonized Africa around the middle to late Miocene with all the four Guinea fowl genera having clearly differentiated by the Pleistocene. Although Ayorinde [54] agrees that Guinea fowls could have evolved from a francolin-like Asiatic ancestor, he suggested that their evolution to modern forms solely occurred in Africa. Recent excavations of the footprint tuffs of the Laetolil beds at Laetoli in Northern Tanzania have revealed the presence of a large variety of footprints from the Pliocene Epoch between 3.5 and 3.8 million years ago [55]. The bird tracks found compare closely with tracks of the living helmeted Guinea fowls common in the Laetoli area today. Guinea fowl remains were also discovered at Shaqadud site in the Sudan around the 4^th^ millennium bp and they do not seem to differ from modern wild specimens [56–58].

Studies show that appearance of Guinea fowls in the history of man's activities is traced to the fifth Egyptian dynasty about 2,400 B.C. when its figure was drawn in a mural [7]. Early domestication is believed to have occurred in Southern Sudan and West Africa [7, 39]. It is also suggested that present day domesticated helmeted Guinea fowls were probably all derived from the West African subspecies* N.m. galeata* [1] which was then repeatedly introduced worldwide [59–61]. According to Crowe [62], wild populations of* N. meleagris* readily become commensals of man, increasing in numbers and distribution because of the water, roosting, and feed resources resulting from human activity. The process of domestication probably continues even now.

Shillington [63] proposed that the languages of Kordofan, west of the middle Nile in Sudan, are linked to the Niger-Congo language family which includes all the Bantu speakers in Africa. This has prompted some linguists and historians to propose that Kordofan in Sudan may have been the original ancestral home of the Niger-Congo language group that then migrated westwards to West Africa. Other linguists, however, feel it might have been the other way round, with Kordofanian being a remote offshoot of Niger-Congo. Shillington [63] also pointed out that by 3000 BCE, the Niger-Congo people had already domesticated Guinea fowls. Based on the Kordofanian proposition, we propose that in the course of their westward expansion into West Africa, the Niger-Congo peoples might have carried along the wild helmeted Guinea fowls and later domesticated them. From West Africa, the Bantu branch of the Niger-Congo expanded southwards and eastwards into Southern, Central, and Eastern Africa. Results from our mtDNA analysis also seem to point to a genetic relationship between West African domesticated helmeted Guinea fowls and most domesticated helmeted Guinea fowls found in Kenya.

Again, it is also imperative to note that the Lugbara, a Nilo-Saharan people of north-western Uganda, have traditionally reared Guinea fowls as one of their main economic activity [64], although information on exactly when it was domesticated is scarce. Considering that the Nilo-Saharan peoples have their roots in Eastern Africa, it is possible that some helmeted Guinea fowl continuously lived in Eastern Africa since antiquity and has been utilized as an economic resource by its people [65]

**Figure 7:**
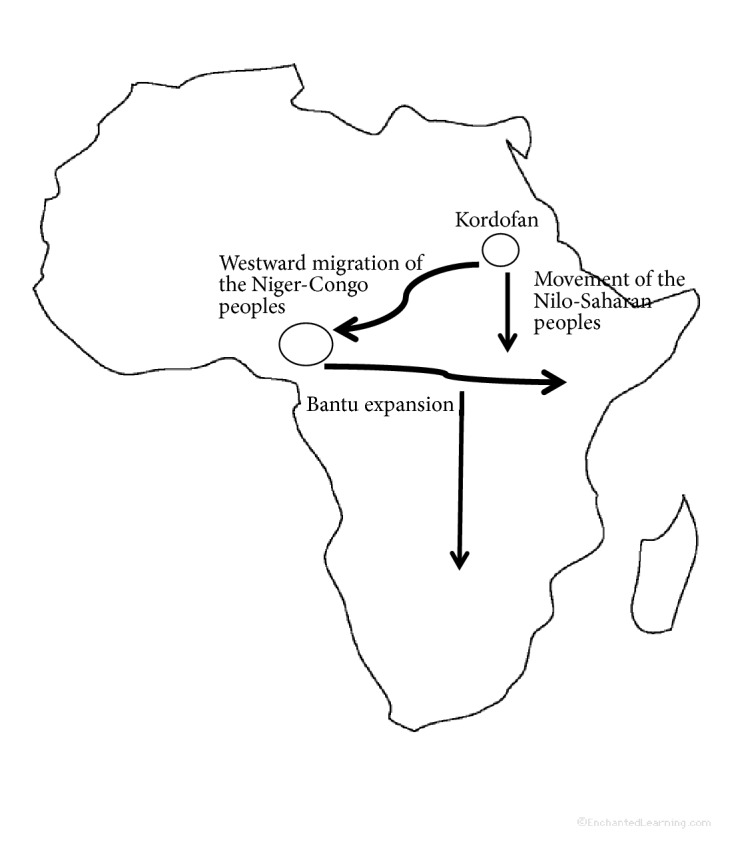
Possible migration routes of the domesticated helmeted Guinea fowls along with the movement of the Niger-Congo and Nilo-Saharan peoples into Kenya (source: http://www.vinotique.com).

Also based on Western Bantu folklore, many Bantu communities of Uganda and Western Kenya claim that their origin is traced to Misri (the present day Egypt), through Congo and Uganda [66]. On the basis of these claims, we then hypothesize that Western Bantus arrived into Uganda and Kenya with the domesticated Guinea fowls (perhaps from Egypt or Sudan). The archaeolinguistic insight into the origin and domestication of helmeted Guinea fowls in Africa is summarized in Figure 7.

## 4. Conclusion

Just like in Nigeria, most domesticated helmeted Guinea fowls in Kenya clustered into two mtDNA haplogroups: HgA and HgB, indicating a genetic relationship between Kenyan and West African Guinea fowls. The wild helmeted Guinea fowls which belong to a different subspecies, are grouped into distinct haplogroups. The lack of a population structure in domesticated helmeted Guinea fowls could suggest intensive genetic intermixing between the domestic populations. The differentiation of the wild Guinea fowls may be due to a clearly distinct demographic history that shaped its genetic profile. All helmeted Guinea fowls in Kenya group into 4 HSP70 haplotypes with two of the haplotypes unique to the wild Guinea fowl. Probably, some of these polymorphisms may be associated with certain environmental adaptations, such as heat tolerance. Analysis of the Kenyan helmeted Guinea fowl population structure and history based on mtDNA variations complimented by archaeological and linguistic insight clearly supports the hypothesis that majority of domesticated helmeted Guinea fowls are related to West African domesticated helmeted Guinea fowls. This study provides initial information on genetic variation across populations of the domesticated and wild helmeted Guinea fowls in Kenya. This is expected to help support the conservation efforts for helmeted Guinea fowls and also develop breeding programs aimed at mitigating the effects of climate change and improving food security.

## Figures and Tables

**Table 1:** Diversity indices of mtDNA D-loop in the helmeted Guinea fowl populations in Kenya.

Population	n	h	k	*π*
Bungoma South	13	0.897±0.067	5.64±2.89	0.0161±0.0093
Teso North	18	0.797±0.090	5.48±2.76	0.0156±0.0088
Bungoma West	18	0.889±0.053	5.26±2.66	0.0150±0.0085
Mt. Elgon	21	0.638±0.079	4.34±2.23	0.0124±0.0071
Wild	20	0.858±0.054	8.39±4.05	0.0238±0.0128

h: haplotype diversity; k: mean number of pairwise differences; *π*: nucleotide diversity.

**Table 2:** Diversity indices of HSP70 gene in the helmeted Guinea fowl populations in Kenya.

Population	n	Number of polymorphic sites	h	k	*π*
Bungoma South	26	0	0.000±0.000	0.000±0.000	0.000±0.000
Teso North	32	1	0.222±0.062	0.222±0.267	0.00048±0.00063
Bungoma West	34	0	0.000±0.000	0.000±0.000	0.000±0.000
Mt. Elgon	42	0	0.000±0.000	0.000±0.000	0.000±0.000
Wild	40	5	0.451±0.051	0.476±0.418	0.00102±0.00099

h: gene diversity; k: mean number of pairwise differences; *π*: nucleotide diversity.

## Data Availability

The sequence data used to support the findings of this study have been deposited in the National Centre for Biotechnology Information (NCBI) sequence read archive (http://www.ncbi.nlm.nih.gov/Traces/) under accession numbers GenBank: MH703540-MH703564 for Guinea fowl mitochondrial DNA haplotypes and GenBank: MH703565-MH703568 for Guinea fowl heat shock protein 70 haplotypes.

## Supplementary Materials

Supplementary Materials Supplementary material accompanies this paper. The following list of figures and tables are included in the Supplementary Materials: (1) Table S1: Genbank accession numbers for mtDNA reference sequences of Nigerian, Kenyan, and Chinese domesticated helmeted Guinea fowls and other published sequences. (2) Table S2: frequencies of mtDNA haplotypes in the helmeted Guinea fowl populations in Kenya. (3) Figure S1: observed and expected distributions of mtDNA pairwise nucleotide differences (mismatches) under the model of growing-declining populations in the helmeted Guinea fowls in Kenya. (4) Table S3: summary of statistics about the demographic history of helmeted Guinea fowl populations in Kenya. (5) Table S4: demographic and spatial expansion of the mtDNA haplotypes in the helmeted Guinea fowl populations in Kenya. (6) Table S5: results from the mtDNA AMOVA of the five helmeted Guinea fowl populations in Kenya. (7) Table S6: results from the mtDNA AMOVA of the wild and domesticated helmeted Guinea fowls in Kenya. (8) Table S7: results from the mtDNA AMOVA of the Teso South and Mt. Elgon, Bungoma West and Bungoma South, and the wild population in Kenya. (9) Table S8: description of the polymorphic sites of the HSP70 haplotypes in the helmeted Guinea fowls in Kenya. (10) Table S9: relative frequencies of HSP70 haplotypes in the helmeted Guinea fowl populations in Kenya. (11) Table S10: result from the HSP70 AMOVA of the wild and domesticated helmeted Guinea fowls in Kenya. (12) Table S11: result from the HSP70 AMOVA of the three groups: Teso South and Mt. Elgon, Bungoma West and Bungoma South, and the wild population of the helmeted Guinea fowls in Kenya.
