# Supplementary material for: Mitochondrial DNA D-Loop Diversity of the Helmeted Guinea Fowls in Kenya and Its Implications on HSP70 Gene Functional Polymorphism
Source: Biomed Res Int. 2018 Nov 13;2018:7314038. doi: 10.1155/2018/7314038 (PMC6258102; doi:10.1155/2018/7314038)
Supplement: Supplementary Materials — Supplementary material accompanies this paper. The following list of figures and tables are included in the Supplementary Materials: (1) Table S1: Genbank accession numbers for mtDNA reference sequences of Nigerian, Kenyan, and Chinese domesticated helmeted Guinea fowls and other published sequences. (2) Table S2: frequencies of mtDNA haplotypes in the helmeted Guinea fowl populations in Kenya. (3) Figure S1: observed and expected distributions of mtDNA pairwise nucleotide differences (mismatches) under the model of growing-declining populations in the helmeted Guinea fowls in Kenya. (4) Table S3: summary of statistics about the demographic history of helmeted Guinea fowl populations in Kenya. (5) Table S4: demographic and spatial expansion of the mtDNA haplotypes in the helmeted Guinea fowl populations in Kenya. (6) Table S5: results from the mtDNA AMOVA of the five helmeted Guinea fowl populations in Kenya. (7) Table S6: results from the mtDNA AMOVA of the wild and domesticated helmeted Guinea fowls in Kenya. (8) Table S7: results from the mtDNA AMOVA of the Teso South and Mt. Elgon, Bungoma West and Bungoma South, and the wild population in Kenya. (9) Table S8: description of the polymorphic sites of the HSP70 haplotypes in the helmeted Guinea fowls in Kenya. (10) Table S9: relative frequencies of HSP70 haplotypes in the helmeted Guinea fowl populations in Kenya. (11) Table S10: result from the HSP70 AMOVA of the wild and domesticated helmeted Guinea fowls in Kenya. (12) Table S11: result from the HSP70 AMOVA of the three groups: Teso South and Mt. Elgon, Bungoma West and Bungoma South, and the wild population of the helmeted Guinea fowls in Kenya. [file 7314038.f1.pdf]

Supplementary information

Table S1: Genbank accession numbers for mtDNA reference sequences of Nigerian, Kenyan and Chinese domesticated helmeted Guinea fowls and other published sequences

Sequence	Accession number	Sequence	Accession number
Isolate_783_KA4	KP218433	Isolate_750_KE1	KP218405
Isolate_781_KA5	KP218432	Isolate_75_LO1	KP218404
Isolate_780_KA5	KP218431	Isolate_749_KE1	KP218403
Isolate_78_LO1	KP218430	Isolate_747_KE5	KP218402
Isolate_746_KE6	KP218401	Isolate_665_KEN1	KP218358
Isolate 745_KE6	KP218400	Isolate_660_KEN1	KP218357
Isolate 744_KE4	KP218399	Isolate_66_AK3	KP218356
Isolate_743_KE1	KP218398	Isolate_659_KEN1	KP218355
Isolate_729_KE6	KP218382	Isolate_65_AK3	KP218354
Isolate_728_KE5	KP218381	Isolate_64_AK2	KP218353
Isolate_727_KE6	KP218380	Isolate 63_AK3	KP218352
Isolate_726_KE6	KP218379	Isolate_62_AK3	KP218351
Isolate_725_KE5	KP218378	Isolate_61_AK3	KP218350
Isolate_724_KE5	KP218377	Isolate_60_AK2	KP218349
Isolate_723_KE5	KP218376	Isolate_6_KAT1	KP218348
Isolate_721_KE4	KP218375	Isolate_59_AK2	KP218347
Isolate_720_KE4	KP218374	Isolate_58_AK2	KP218346
Isolate_72_AK4	KP218373	Isolate_57_AK2	KP218345
Isolate_719_KE3	KP218372	Isolate_56_AK2	KP218344
Isolate_724_KE5	KP218377	Isolate_60_AK2	KP218349
Isolate_723_KE5	KP218376	Isolate_6_KAT1	KP218348
Isolate_721_KE4	KP218375	Isolate_59_AK2	KP218347
Isolate_720_KE4	KP218374	Isolate_58_AK2	KP218346
Isolate_72_AK4	KP218373	Isolate_57_AK2	KP218345
Isolate_719_KE3	KP218372	Isolate_56_AK2	KP218344
Isolate_717_KE2	KP218371	Isolate_55_AK2	KP218343
Isolate_716_KE1	KP218370	Isolate_54_AK1	KP218342
Isolate_714_KE2	KP218369	Isolate_53_AK1	KP218341
Isolate_713_KE1	KP218368	Isolate_52_AK1	KP218340
Isolate_712_KE2	KP218367	Isolate_51_AK1	KP218339
Isolate_711_KE1	KP218366	Isolate_50_TA5	KP218338
Isolate_71_AK4	KP218365	Isolate_5_KAT	KP218337

**Sequence**	**Accession number**	**Sequence**	**Accession number**
Isolate_70_AK4	KP218364	Isolate 495_TA5	KP218336
Isolate_7_KAT1	KP218363	Isolate_493_TA5	KP218335
Isolate_69_AK2	KP218362	Isolate_492_TA5	KP218334
Isolate_717_KE2	KP218371	Isolate_55_AK2	KP218343
Isolate_713_KE1	KP218368	Isolate_52_AK1	KP218340
Isolate_712_KE2	KP218367	Isolate_51_AK1	KP218339
Isolate_711_KE1	KP218366	Isolate_50_TA5	KP218338
Isolate_71_AK4	KP218365	Isolate_5_KAT	KP218337
Isolate_70_AK4	KP218364	Isolate 495_TA5	KP218336
Isolate_7_KAT1	KP218363	Isolate_493_TA5	KP218335
Isolate_69_AK2	KP218362	Isolate_492_TA5	KP218334
Isolate_102_AK5	KP218267	isolate_734_KE2	KP218388
Isolate_101_AK5	KP218266	isolate_733_KE2	KP218387
Isolate_100_AK5	KP218265	isolate_732_KE2	KP218386
Isolate_10_KAT1	KP218264	isolate_731_KE2	KP218385
Isolate_1_KAT1	KP218263	isolate_730_KE5	KP218384
Isolate_742_KE3	KP218397	isolate_73_LO1	KP218383
Isolate 741_KE1	KP218396	Isolate_19546_CH	KP218287
Isolate_33_TA5	KP218315	Isolate_19545_CH	KP218286
Isolate_32_TA4	KP218314	Isolate_19544_CH	KP218285
Isolate_30_TA1	KP218313	Isolate_19543_CH	KP218284
Isolate_3_KAT1	KP218312	Isolate_19541_CH	KP218283
N.m. meleagris	NC_006382	*Acrylium vulturinum*	NC_014180

Table S2: Frequencies of mtDNA haplotypes in the helmeted Guinea fowl populations in Kenya

Haplotype	Bungma South	Teso North	Bungoma West	Mt. Elgon	Wild
Hap1	0	0	1	0	0
Hap2	2	0	4	2	0
Hap3	0	0	1	0	0
Hap4	4	8	5	13	0
Hap5	0	0	2	0	0
Hap6	0	1	1	0	0
Hap7	1	0	1	4	0
Hap8	2	3	1	0	0
Hap9	1	1	1	1	0
Hap10	0	0	1	0	0
Hap11	1	1	0	1	0
Hap12	0	1	0	0	0
Hap13	0	1	0	0	0
Hap14	1	1	0	0	0
Hap15	0	1	0	0	0
Hap16	1	0	0	0	0
Hap17	0	0	0	0	2
Hap18	0	0	0	0	6
Hap19	0	0	0	0	1
Hap20	0	0	0	0	2
Hap21	0	0	0	0	1
Hap22	0	0	0	0	5
Hap23	0	0	0	0	1
Hap24	0	0	0	0	1
Hap25	0	0	0	0	1
n	13	18	18	21	20

n= number of individuals

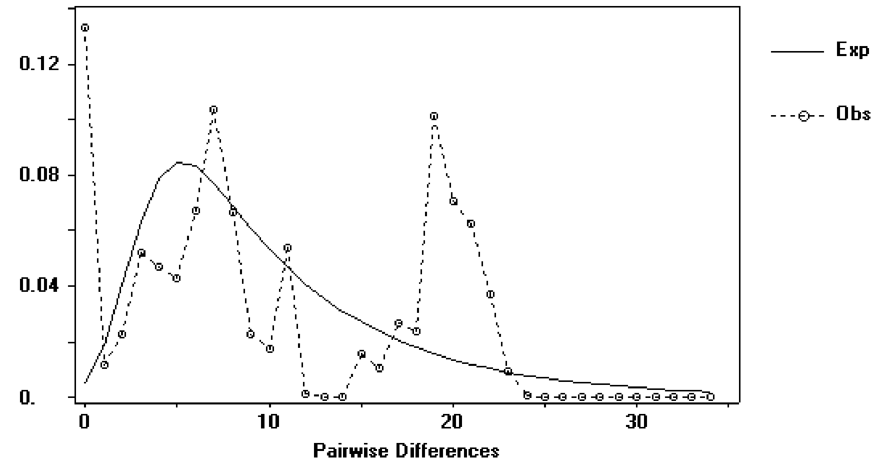

Figure S1: Observed and expected distributions of mtDNA pair-wise nucleotide differences (mismatches) under the model of growing-declining populations in the helmeted Guinea fowls in Kenya

Table S3: Summary of statistics about the demographic history of helmeted Guinea fowl populations in Kenya

Population	SSD (P-value)	D (P-value)	F_s (P-value)
Bungoma South	0.019 (0.486)	1.43 (0.942)	-0.39 (0.446)
Teso North	0.041 (0.236)	1.68 (0.959)	-0.13 (0.455)
Bungoma West	0.053 (0.031)*	1.46 (0.943)	-1.11 (0.317)
Mt. Elgon	0.145 (0.075)	0.99 (0.860)	3.05 (0.915)
Wild	0.040 (0.446)	0.15 (0.654)	1.71 (0.807)

SSD= sum of squared differences; D= Tajima's statistics; F_s= Fu's statistics; *P<0.05

Table S4: Demographic and spatial expansion of the mtDNA haplotypes in the helmeted Guinea fowl populations in Kenya

	Demographic expansion		Spatial expansion	
Population	Raggedness index	Raggedness p value	Raggedness index	Raggedness p value
Bungoma South	0.0559	0.3860	0.0559	0.6770
Teso North	0.0986	0.1890	0.0986	0.5680
Bungoma West	0.1203	0.0210	0.1203	0.2570
Mt. Elgon	0.2877	0.0110	0.2877	0.6440
Wild	0.0753	0.1860	0.0753	0.3860

Table S5: Results from the mtDNA AMOVA of the five helmeted Guinea fowl populations in Kenya

Source of variation	df	Sum of squares	Variance components	Percentage variation	F_{ST}	P-value
Among populations	4	230	3.05	51.5	0.515	0.00
Within populations	85	244	2.87	48.5		

df= degrees of freedom

Table S6: Results from the mtDNA AMOVA of the wild and domesticated helmeted Guinea fowls in Kenya

Source of Variation	df	Sum of squares	Variance components	Percentage variation	F_{ST}	P-value
Among populations	1	220	6.99	70.7	0.707	0.00
Within populations	88	254	2.89	29.3		

df= degrees of freedom

Table S7: Results from the mtDNA AMOVA of the Teso South and Mt. Elgon, Bungoma West and Bungoma South, and the wild population in Kenya

Source of Variation	df	Sum of squares	Variance components	Percentage variation	F_{ST}	P-value
Among populations	2	226	2.79	57.0	0.570	0.00
Within populations	87	249	2.86	43.0		

df= degrees of freedom

Table S8: Description of the polymorphic sites of the HSP70 haplotypes in the helmeted Guinea fowls in Kenya

	Position (in bp)		
Haplotype	42	60	264
TGC	T	G	C
TAC	T	A	C
TGT	T	G	T
CGC	C	G	C

A: adenine; G: guanine; C: cytosine; T: thymine

Table S9: Relative frequencies of HSP70 haplotypes in the helmeted Guinea fowl populations in Kenya

Haplotype	Bungoma South	Teso North	Bungoma West	Mt. Elgon	Wild
TGC	1.00	0.875	1.00	1.00	0.70
TAC	0.00	0.125	0.00	0.00	0.00
TGT	0.00	0.00	0.00	0.00	0.25
CGC	0.00	0.00	0.00	0.00	0.05

A: adenine; G: guanine; C: cytosine; T: thymine

Table S10: Result from the HSP70 AMOVA of the wild and domesticated helmeted Guinea fowls in Kenya

Source of variation	df	Sum of squares	Variance components	Percentage variation	P-value
Among populations	1	4.06	0.319	29.3	0.00
Among individuals within population	172	22.6	0.0542	49.6	
Within individuals	174	4.00	0.230	21.1	

df= degrees of freedom

Table S11: Result from the HSP70 AMOVA of the three groups; Teso South and Mt. Elgon, Bungoma West and Bungoma South, and the wild population of the helmeted Guinea fowls in Kenya

Source of variation	df	Sum of squares	Variance components	Percentage variation	P-value
Among populations	2	4.25	0.0177	18.7	0.00
Among individuals within population	171	22.4	0.0539	57.0	
Within individuals	174	4.00	0.0230	24.3	

df= degrees of freedom
